# Dissecting the METTL3/STC2 axis in colorectal cancer: implications for drug resistance and metastasis

**DOI:** 10.1007/s10565-025-10043-5

**Published:** 2025-06-10

**Authors:** Qiang Su, Kaiyue Wang, Ruohan Liao, Hanyu Zhang, Bochu Wang

**Affiliations:** 1 Key Laboratory of Biorheological Science and Technology, Ministry of Education, College of Bioengineering, Chongqing University, Chongqing, 400030 China; 2 Department of Pharmacy, Nanchong Central Hospital, The Second Clinical Medical College, North Sichuan Medical College, Nanchong, Sichuan China; 3 School of Pharmacy, North Sichuan Medical College, Nanchong, Sichuan China; 4 Nanchong Key Laboratory of Individualized Drug Therapy, Nanchong, Sichuan China

**Keywords:** METTL3, STC2, M6 A modification, Colorectal cancer, Drug resistance

## Abstract

In recent years, the role of epigenetic modifications, especially N6-methyladenosine (m6A) modifications, in the occurrence and development of cancer has received increasing attention. This study aims to elucidate the role of m6A modification in colorectal cancer (CRC), focusing on the effect of METTL3 on STC2 expression and its effects on cell proliferation, drug resistance and metastasis. Using MeRIP-seq, mRNA-seq, EdU staining, CCK-8 (Cell Counting Kit-8) assay, Transwell assay, Western blot and flow cytometry, this study confirmed that RNA methylation was predominantly located in the CDS region and that STC2 was overexpressed in advanced cancer and 5-FU (5-Fluorouracil)-resistant cell lines. Knockdown of STC2 increased the sensitivity of cells to 5-FU, reduced cell proliferation and metastatic capacity, and indicated that METTL3 positively regulates STC2 m6A modification. Further experiments showed that METTL3 knockdown reduced the IC50 (Half Maximal Inhibitory Concentration) of 5-FU-resistant CRC cells, inhibited cell proliferation, ERS (Endoplasmic Reticulum Stress) and oxidative stress, and reduced KRAS G12 and G13 mutations, and these effects were reversed by STC2 overexpression. In vivo, METTL3 knockdown enhanced the efficacy of 5-FU and inhibited tumor metastasis, whereas STC2 overexpression counterbalanced these benefits. Overall, our findings suggest the METTL3/STC2 axis as a promising therapeutic target to combat drug resistance and metastasis in colorectal cancer.

**Supplementary Information:**

The online version contains supplementary material available at 10.1007/s10565-025-10043-5.

## Introduction

Colorectal cancer (CRC) is a malignant tumor that occurs in the colon and rectum, which are the main components of the large intestine (Yuan et al. 2025a, b). CRC has multiple etiologies, including poor lifestyle, environment, diet, and genetics (e.g., adenoma, inflammatory bowel disease, etc.) (Yuan et al. 2025a, b). Besides, various chemicals, parasites and chronic gastrointestinal diseases are also potential triggers for the development of CRC (Mauri et al. 2025). Based on the accumulation of multiple oncogene/oncogene mutations, colorectal cell deterioration results from multiple steps of interaction (Zhou, Zhang et al. 2024). Due to the lack of early symptoms and detection markers, CRC is often diagnosed at an advanced stage, limiting curative treatment options like surgery (Mauri et al. 2025). Therefore, there is an urgent need to investigate the therapeutic mechanisms of CRC.

Chemotherapy is the key therapy for mid- to late-stage CRC, and commonly used chemotherapeutic agents include 5-fluorouracil (5-FU), but drug accumulation can induce drug-resistant gene expression in tumor cells, leading to chemotherapy resistance (Dong et al. 2024). Mutations in the KRAS gene are among the most common mutations in cancer, particularly in RAS-driven cancers, where KRAS mutations play a important role (Fu et al. 2025). G12 and G13 are two mutation sites of KRAS that play an important role in tumor resistance, which may lead to sustained activation of KRAS protein and affect other signaling pathways, thereby promoting tumor development and drug resistance (Thongyoo et al. 2025). Study has found that DNA damage repair and KRAS mutations are closely related (Vokes et al. 2023). For example, in KRAS-mutant pancreatic ductal adenocarcinoma (PDAC), inhibition of DNA damage repair can significantly enhance the drug sensitivity of KRAS-mutant PDAC (Klomp et al. 2021). Furthermore, KRAS mutations can also enable tumor cells to evade cellular damage and apoptosis induced by chemotherapy-triggered oxidative stress, thereby maintaining cellular homeostasis (DeNicola et al. 2011). However, in colorectal cancer, the relationship connecting chemotherapy-induced cellular damage and repair, oxidative stress, and cellular drug resistance remains unclear.

N6-methyladenosine (m6 A) modifications are the most frequent epigenetic modifications in eukaryotic RNAs, which can ensure timely and accurate gene expression (Kang et al. 2024). m6 A modification is regulated by methyltransferases (METTL3/14, WTAP, and KIAA1429), demethylases (FTO and ALKHB5) and methylation recognition proteins (YTHDF1, YTHDF2 and YTHDF3 (Lin et al. 2024). Meanwhile, there is a close association between 5-FU resistance and m6 A modification in clinical cancer therapy (Kang et al. 2025). Emerging research suggests that m6 A modification may drive chemoresistance by regulating tumor DNA damage repair and mutation accumulation (Mamontova et al. 2024). In non-small cell lung cancer, global m6 A methylation levels positively correlate with KRAS mutation status. Further experiments have demonstrated that m6 A methylation enhances the stability of DNA repair-related genes, thereby promoting post-chemotherapy genomic recovery and platinum drug resistance (Yu et al. 2025). As a first-line chemotherapeutic agent for CRC, 5-FU resistance remains a major clinical challenge. However, it remains unclear whether m6 A modification contributes to 5-FU resistance in CRC by modulating tumor mutagenesis and DNA damage repair pathways. This knowledge gap warrants systematic exploration.

STC2 (Stanniocalcin-2) is a glycoprotein that induces cellular response to stress to prevent apoptosis and is widely upregulated in human tumors (Jiang et al. 2022). STC2 attributes to the development of acquired resistance to chemotherapy and radiotherapy (Cheng et al. 2018). Recent study denoted that STC2 overexpression in CRC can lead to poor prognosis and accelerate CRC cell proliferation and metastasis (Ke et al. 2019). In addition, STC2 expression is significantly upregulated in response to endoplasmic reticulum stress (ERS), hypoxia, and nutrient deficiency, thereby enhancing cellular adaptability and inhibiting apoptosis (Qie and Sang 2022). Under ERS, the homeostasis of intracellular calcium ions is unbalanced, which promotes oxidative stress, and then causes excessive production of ROS, which can directly attack DNA, cause DNA damage, and activate apoptosis pathway. In order to escape apoptosis, tumor cells may up-regulate proteins related to DNA damage repair pathway, leading to genomic instability and promoting the process of cancer (Biryukov, Semenov et al. 2023). The intestinal epithelium has a rich endoplasmic reticulum structure and ERS is associated with CRC development(Stengel et al. 2020). Studies have shown that STC2 ameliorates ataxin-3 and huntingtin-mediated ERS and cell death (Wahlstrom 2009). Mutations in the KRAS protein may lead to tumor progression and drug resistance. The established roles of METTL3 in m6 A methylation and STC2 in CRC stress response and drug resistance highlight the potential METTL3/STC2 axis, regulated via m6 A modification, as a key determinant of chemoresistance. Therefore, we hypothesized that STC2 m6 A modification may alter ERS by modulating KRAS mutation in tumor cells and participate in CRC resistance.

In our study, we explored the effects on ERS and oxidative stress, DNA damage and KRAS mutation in CRC cells by revealing the mechanism of action of the METTL3/STC2 axis, and whether it further affected the proliferation, metastasis and resistance to 5-FU in CRC cells. A key novelty of this study lies in uncovering the METTL3-mediated m6 A modification as a direct regulatory mechanism for STC2, thereby establishing METTL3/STC2 axis crucial for conferring 5-FU resistance in CRC, specifically through modulating ERS, oxidative stress, and KRAS mutations. This study is expected to reveal improved understanding of the mechanism of 5-FU-resistant CRC, contribute to the development of new methods to overcome drug resistance.

## Methods

### Tissue samples

CRC tissues and para-carcinoma tissues were collected from CRC patients diagnosed in Nanchong Central Hospital from Jan. 2019 to Dec. 2022. The study protocol strictly adhered to the ethical guidelines of the Declaration of Helsinki. Prior to sample collection, all participants received a detailed explanation of the study purpose, potential risks/benefits, and data usage. Written informed consent was obtained from each patient after ensuring their full understanding of voluntary participation and the right to withdraw at any stage without affecting clinical treatment. In this experiment, we collected cancerous tissue removed during surgery from 46 patients after diagnosis and collected adjacent non-cancerous tissue as paired control samples. To protect patient confidentiality, all samples were anonymized immediately after collection by replacing personal identifiers with unique coding numbers. Clinical data were securely stored in password-protected databases with restricted access limited to authorized researchers. Samples were stored in liquid nitrogen immediately after collection. This study had also been approved by the Ethics Committee of Nanchong Central Hospital (Approval No. 2023–041).

### Establishment of 5-FU resistant cell lines

RKO and HCT116/5-FU-resistant cell lines were established using repeated high-dose shocks combined with stepwise increases in 5-FU concentration (1 → 2 → 4 → 8 → 16 μM, each maintained for 2 weeks over a total period of 10 weeks). Parental cells (RKO and LOVO, 5 × 10^5^ cells/mL) were inoculated in 6-well plates. After attachment, the medium was replaced with Dulbecco’s Modified Eagle Medium containing 10% FBS and 1 μg/mL 5-FU. Following 48 h of treatment, cells were washed with D-Hank’s solution and cultured in drug-free medium until 80% confluency. This cycle of 5-FU exposure (1 μg/mL, 48 h) and recovery was repeated 3 times to achieve stable proliferation in 1 μg/mL 5-FU. Subsequently, the 5-FU concentration was incrementally doubled every 2 weeks, with cell viability monitored by CCK-8 (> 80% survival required for progression). Resistance was validated by IC50 assays (CCK-8) showing a > 15-fold increase in 5-FU resistance compared to parental cells.

### In vivo xenograft modeling

BALB/c-nu nude mice (6-week old, weight 18–20 g) were purchased and housed in the North Sichuan Medical College Laboratory Animal Center. All animals were trained in SPF conditions and had free access to sterile food and water. A total of 60 BALB/c-nu nude mice were used for the in vivo xenograft model experiments in this study, randomly allocated into different treatment groups with 6 mice per group (*N* = 6). The nude mice were subcutaneously injected with 5-FU resistant RKO cells (2.0 × 10^6^). All mice were received a 5-FU injection. Afterwards, the tumor size of nude mice was observed at 7, 14, 21 and 28 days. Animal experiments were conducted in compliance with the ARRIVE guidelines. Animal experiments were approved by the Ethics Committee of North Sichuan Medical College. Tumor burden and animal welfare were monitored daily, and humane endpoints were strictly enforced (tumor volume ≤ 1,500 mm^3^; body weight loss ≤ 20%). After 28 d, the nude mice were killed, and the tumor was collected and weighed.

### Statistical analysis

Statistical analyses were performed using Graphpad Prism V10. Data are presented as mean ± SD. Comparisons between two groups were performed using an unpaired, two-tailed Student’s T-test. Comparisons among three or more groups were performed using ANOVA followed by Tukey's multiple comparisons test. A *P*-value < 0.05 was considered statistically significant. All in vitro experiments were conducted with at least three independent biological replicates. For detailed information regarding the methodology, please refer to Supplementary file #1.

## Results

### Identification and Functional Analysis of m6 A-Modified Genes in CRC

**Fig. 1:**
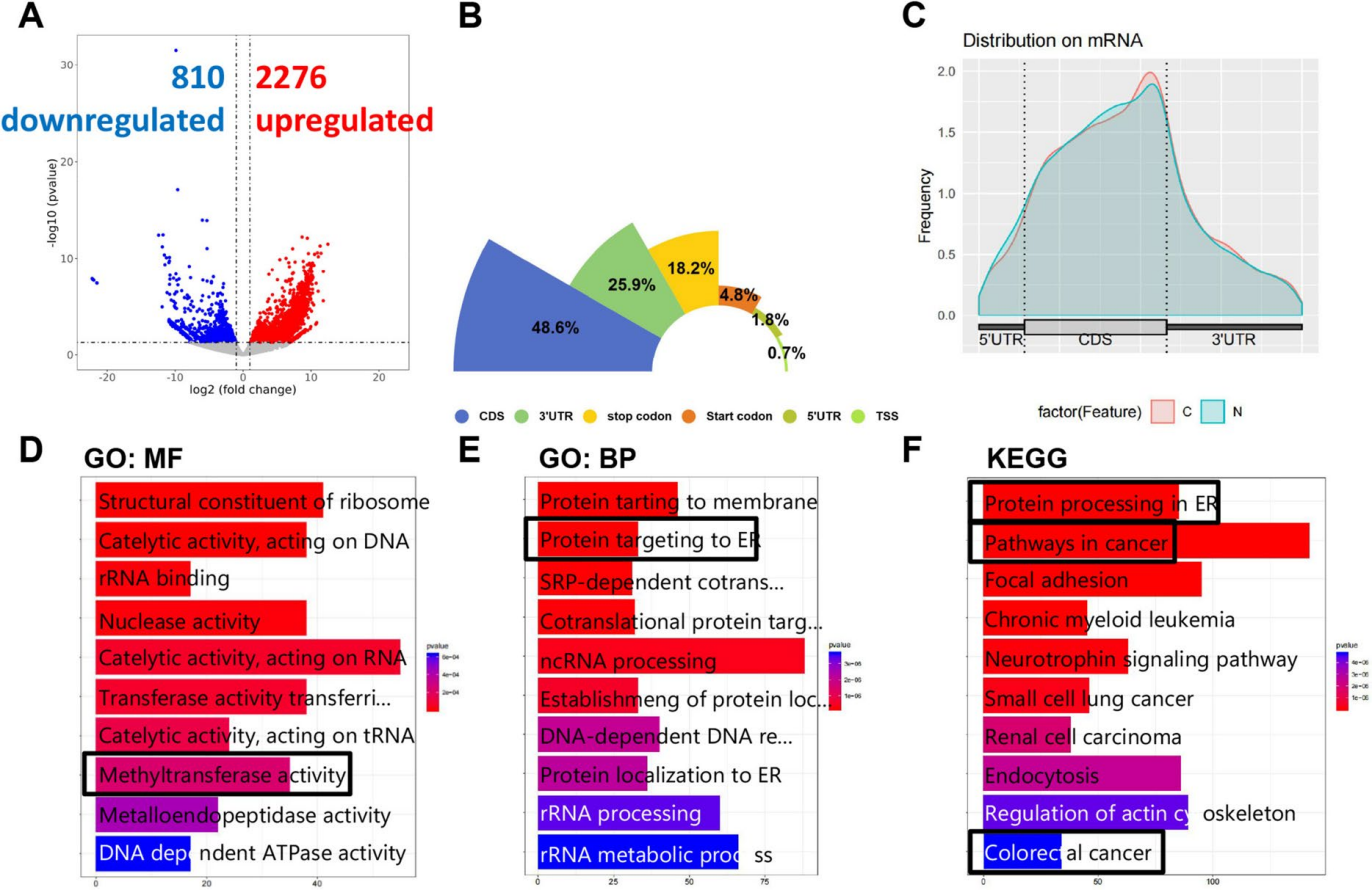
Comprehensive analysis of m6A modification in CRC. (**A**) mRNA-seq identified 2276 upregulated genes and 810 downregulated genes. (**B**, **C**) meRIP-seq showing RNA methylation mainly in the CDS region (48.6%), and 3’UTR (25.9%) (**D**) GO analysis indicating association with methyltransferase activity. (**E**) GO analysis showing connection with endoplasmic reticulum proteins. (**F**) Gene set association with CRC and endoplasmic reticulum

To explore the mechanism of m6 A modification in CRC, we first conducted analyses using meRIP-seq and mRNA-seq. The mRNA-seq analysis revealed 2276 significantly upregulated genes and 810 significantly downregulated genes (Fig. 1A). Integration with meRIP-seq results indicated that RNA methylation primarily occurred in the CDS region, accounting for 48.6%, with 3’UTR and 5’UTR accounting for 25.9% and 1.8%, respectively (Fig. 1B, C). Collectively, further GO and KEGG enrichment analyses provided initial insights into the functional relevance of genes with altered m6 A modification and expression in CRC. Notably, the enrichment of terms such as'Methyltransferase activity’ directly supported our investigation into the role of m6 A regulatory enzymes (Fig. 1D). Furthermore, the significant enrichment of pathways related to the endoplasmic reticulum, including'Protein targeting to ER'and'Protein processing in ER', strongly suggested a potential link between m6 A modification and ER function or stress in CRC (Fig. 1E, F). This is further underscored by the enrichment of general'Pathways in cancer'and specifically'Colorectal cancer', reinforcing the relevance of these identified genes and pathways to the disease context under investigation (Fig. 1F).

### Identification of STC2 as a key gene in CRC.

**Fig. 2:**
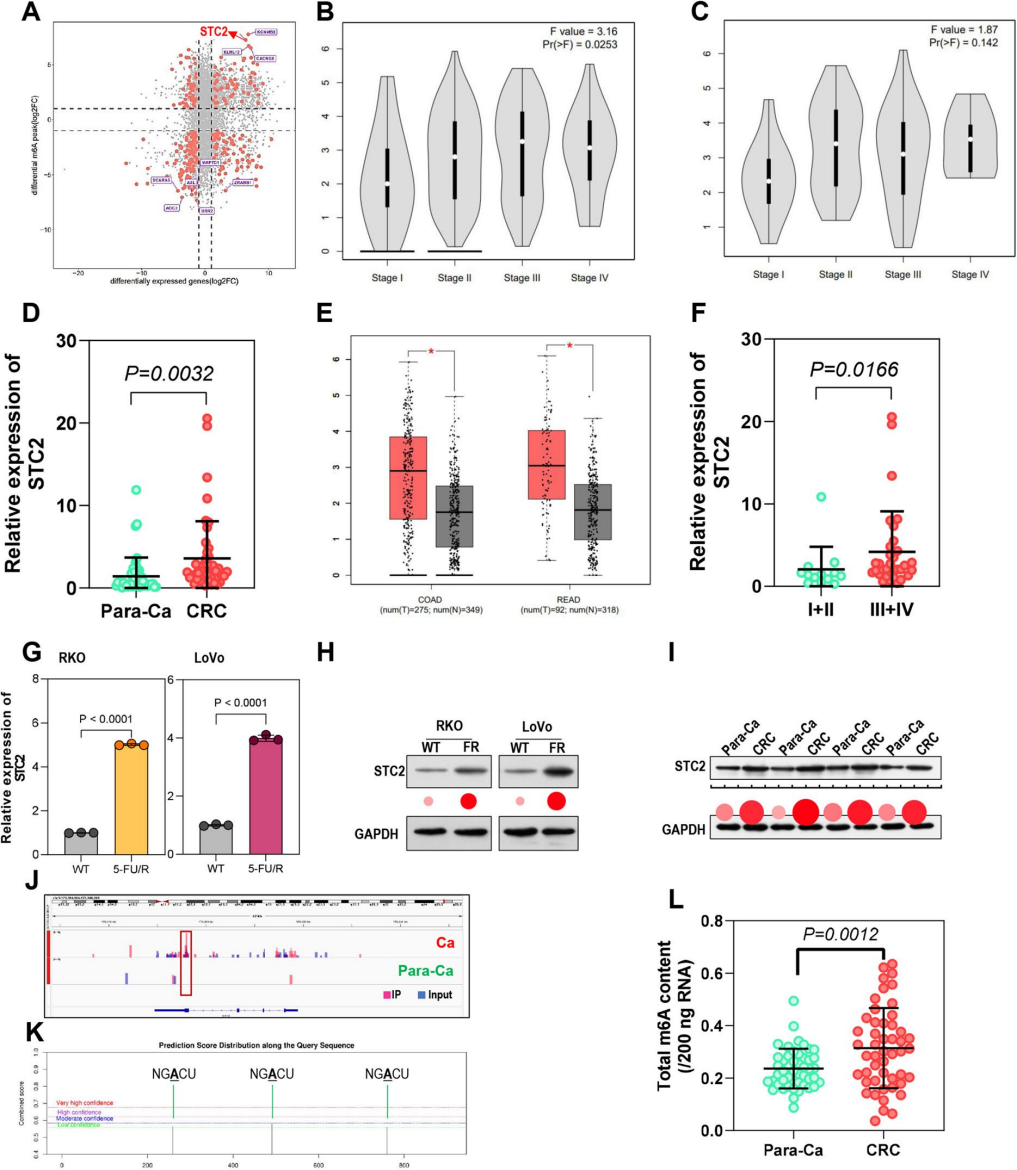
Identification of STC2 as a key gene in CRC. (**A**) Identification of key genes including STC2, KCNMB3, KLHL13, and CACNG8 with high expression level and high m6A methylation. (**B**, **C**) GEPIA database shows increasing STC2 expression with tumor stage progression. (**D**) qPCR showing higher STC2 expression in cancer tissues than adjacent normal tissues. (**E**) Confirmation of high STC2 expression in cancer tissues using GEPIA database. Colorectal Adenocarcinoma (COAD), Rectal Adenocarcinoma (READ). (**F**) Lower STC2 expression in early-stage compared to late-stage CRC. (**G**, **H**) STC2 upregulation in 5-FU resistant cell lines. (**I**) Western blot showing STC2 protein differences in clinical tissues. (**J**) SRAMP database revealing three modification sites on STC2 mRNA. (**K**) IGV map indicating lower m6A levels in the STC2 CDS region in adjacent normal tissues compared to cancer tissues. (**L**) Higher m6A levels in cancer tissues compared to adjacent normal tissues. *p < 0.05, **p < 0.01

To identify candidate factors from the meRIP-seq data, four genes (STC2, KCNMB3, KLHL13, and CACNG8) were selected because they displayed both significant m6 A hypermethylation and high expression (Fig. 2A). The GEPIA database showed that STC2 expression significantly increased with tumor stage progression (Figure S1, Fig. 2B, C). In clinical tissue samples, qPCR confirmed a significant upregulation of STC2 expression in tumor tissues relative to adjacent normal counterparts from CRC patients (Fig. 2D, Table 1), which was confirmed by GEPIA database analysis (Fig. 2E). Further analysis revealed that STC2 was significantly lower in early-stage CRC compared to late-stage (Fig. 2F). STC2 expression was highest in LoVo and RKO cells among the FHC and colorectal cancer cell lines examined. Thus, LoVo and RKO cell lines were utilized for subsequent experiments. Besides, we found that STC2 expression was significantly upregulated in 5-FU resistant RKO and LoVo cell lines (Fig. 2G, H), and western blot analysis of clinical tissues revealed noticeable differences at the protein level (Fig. 2I). The IGV map based on MeRIP-seq data showed that the m6 A modification level in the STC2 CDS region was downregulated in adjacent normal tissues compared to cancer tissues (Fig. 2J). To further understand the modification mechanism of STC2, we analyzed the STC2 sequence using the SRAMP database, which revealed three modification sites on the STC2 mRNA (Fig. 2K). Furthermore, m6A levels were found to be significantly higher in CRC tissues compared to adjacent normal tissues (Fig. 2L). In conclusion, m6 A modification of STC2 may play a crucial role in CRC.

**Table 1:** Clinicopathological features of CRC patients accoriding to STC2 expression

Feature		Expression of STC2 (N)		*p* value
		Low expression	High expression	
**Age**				*0.5499*
	≤ 60	11	8	
	> 60	12	15	
**Sex**				*0.3726*
	Male	12	8	
	Female	11	15	
**Location**				*0.7672*
	Colon	11	10	
	Rectum	12	13	
**Differentiation**				*0.0093*
	Poor	22	15	
	Moderate/Well	1	8	
**CEA status**				*0.0032*
	Normal	16	6	
	Elevated	7	17	
**T stage**				*0.0182*
	T1-T2	16	8	
	T3-T4	7	15	
**M stage**				*0.2343*
	M0	15	11	
	M1	8	12	
**​N stage​**				*0.0025*
	N0	14	4	
	N1-N2	9	19	
**TNM stage**				*0.0219*
	I + II	10	3	
	III + IV	13	20	

### STC2 induced the proliferation and ERS in 5-FU-resistant CRC cells

**Fig. 3:**
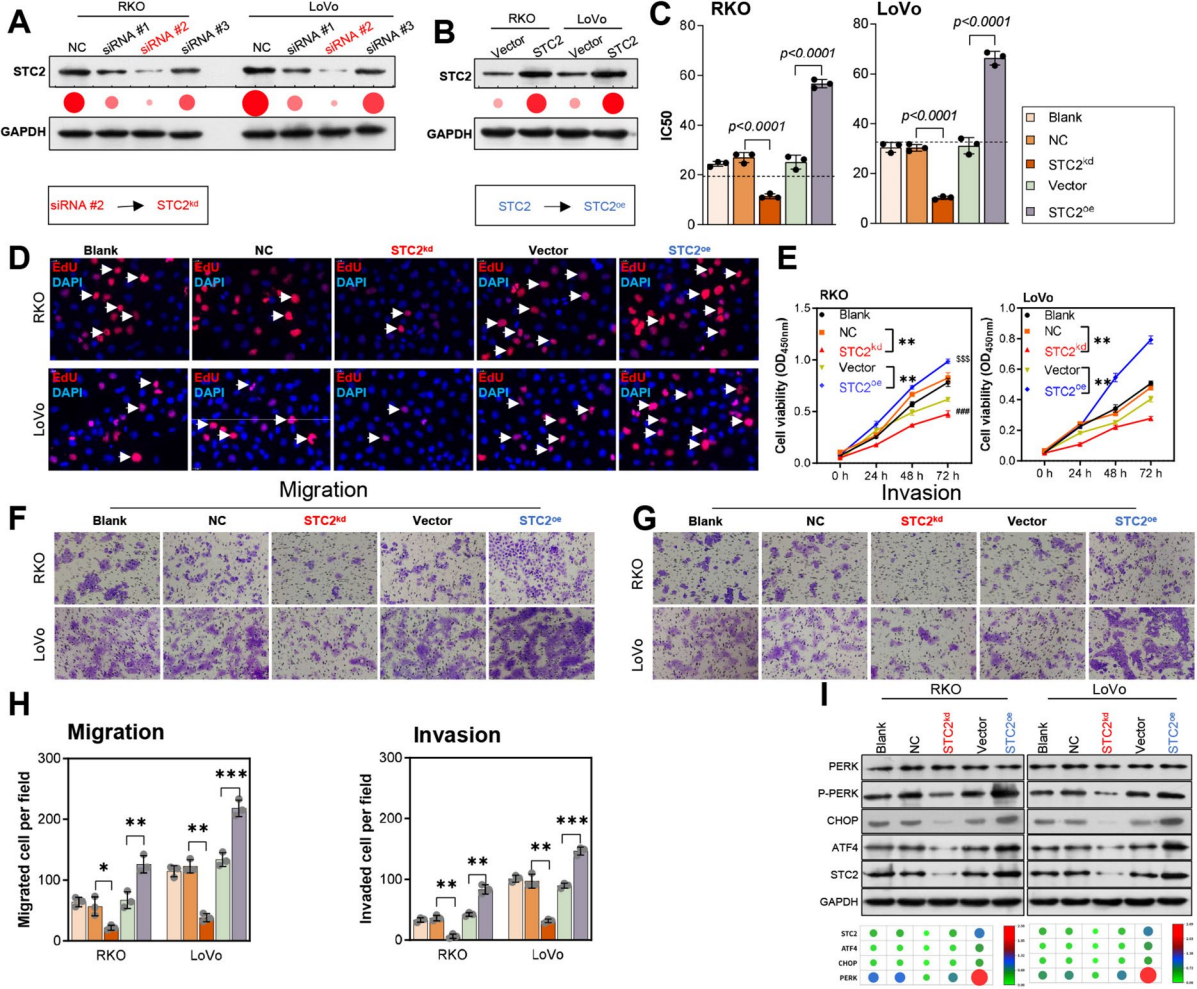
STC2 regulates cell proliferation and ERS in 5-FU-resistant CRC cells. (**A**) siRNA #2 had highest knockdown efficiency for STC2. (**B**) STC2 expression in RKO and LoVo cell was confirmed by western blotting. (**C**) Knockdown of STC2 increased cell sensitivity to 5-FU. (**D**, **E**) EdU staining showing reduced cell proliferation with STC2 silencing, increased with STC2 overexpression. (**F**, **G**, **H**) Transwell assay showing enhanced cell migration and invasion by STC2 overexpression, suppressed by knockdown. (**I**) ERS-related protein expression changes by western blotting. **p < 0.01, ***p < 0.001

To further verify the influence of STC2 on the related functions of 5-FU resistant CRC cells, siRNA targeting STC2 and overexpression vector of STC2 was involved to regulate the expression of STC2 in CRC cells artificially. Accordingly, siRNA #2 had the highest knockdown efficiency of STC2 in cells, and the STC2 overexpression vector had a significant upregulation effect on RKO and LoVo cell lines (Fig. 3A, B). Interestingly, the experiment revealed that STC2 knockdown facilitated an increased sensitivity of cells to 5-FU, while STC2 exhibited a significant role in promoting cellular resistance to 5-FU (Fig. 3C). EdU staining results denoted that STC2 silencing dramatically reduced cell proliferation activity, while overexpression of STC2 notably enhanced cell proliferation activity in 5-FU resistant CRC cells (Fig. 3D). Then CCK-8 data signified that cell viability was observably decreased in STC2-silenced 5-FU resistant CRC cells, while markedly increased in STC2-overexpressed 5-FU resistant CRC cells (Fig. 3E). Meanwhile, Transwell assays results showed that STC2 overexpression and knockdown significantly enhanced and suppressed cell migration and invasion, respectively (Fig. 3F, G, H). Based on the GO and KEGG pathway that showed in Fig. 1, endoplasmic reticulum related protein was included in it. Therefore, ERS -related proteins, PERK, ATF4 were measured. Results showed that ERS-related proteins’ expression were increased in STC2-overexpressed cells. Conversely, the proteins’ expression was suppressed when STC2 was knockdown **(**Fig. 3I).

### STC2 silencing inhibited the resistance to 5-FU in vivo

**Fig. 4:**
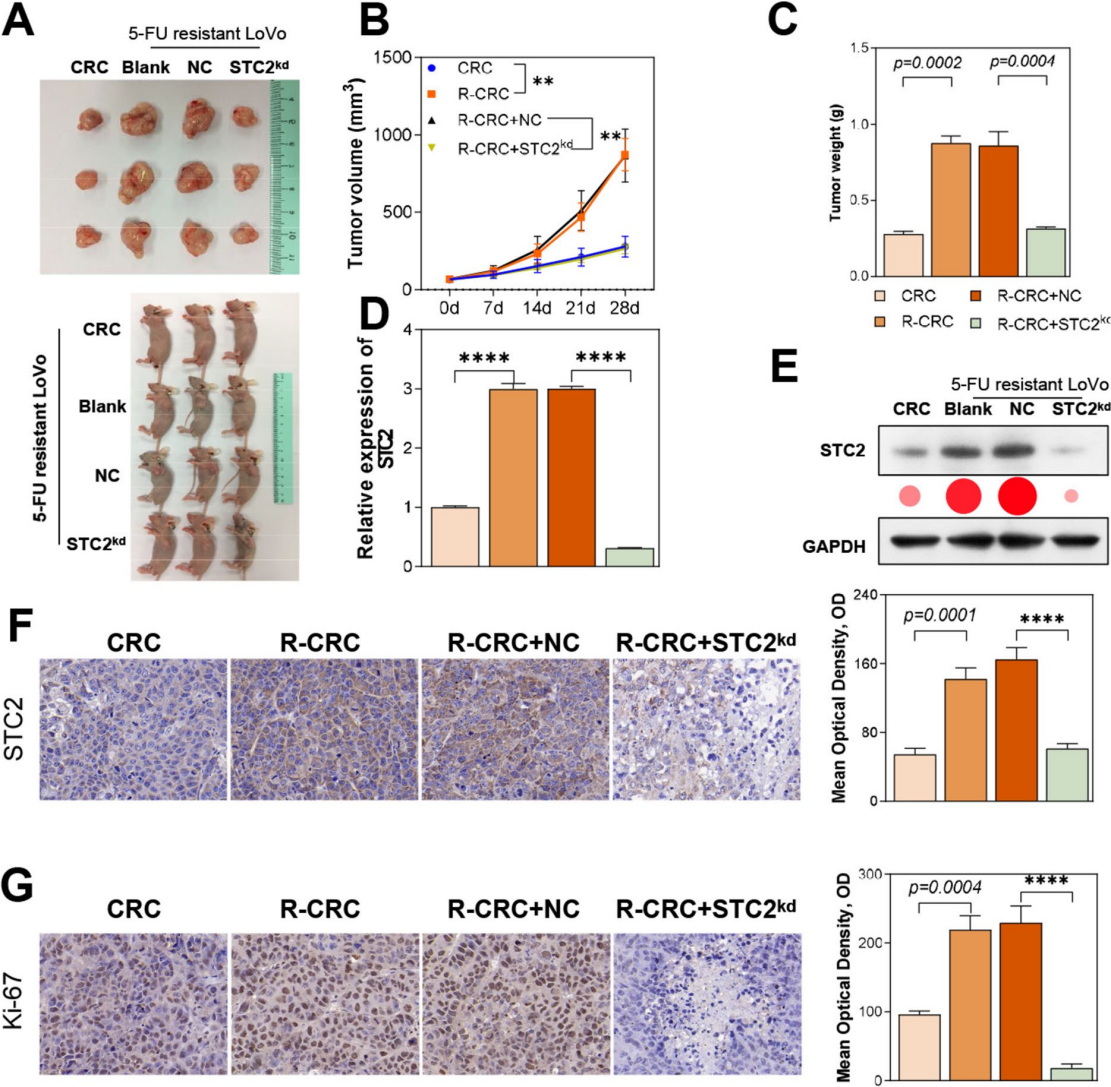
STC2 silencing increased sensitivity to 5-FU and reduced tumor growth in vivo. (**A**, **B**, **C**) STC2 knockdown enhanced the inhibitory effect of 5-FU on tumor growth by tumor bearing experiments in nude mice. (**D**, **E**) qPCR, Western blot was used to confirm STC2 expression levels in tumors. (**F**) Ki-67 and STC2 expression was detected using IHC in tumors. ***p < 0.001 vs. CRC, ^$$^*p* < 0.01, ^$$$^*p* < 0.001, vs. R-CRC + NC

To elucidate the sensitivity of STC2 to 5-FU in vivo, a subcutaneous tumor-bearing model was established to explore this issue. As shown in Fig. 4A, 5-FU significantly inhibited the growth of subcutaneous tumors established by LoVo wild-type cells in nude mice. However, for 5-FU resistant strains, the influence of 5-FU on their growth was minimal. In STC2-knockdown LoVo tumor-bearing mice, there was an increased sensitivity to 5-FU (Fig. 4A, B) and a significant reduction in tumor weight (Fig. 4C). Further qPCR, Western blot, and immunohistochemistry experiments confirmed the expression levels of STC2 in the tumors (Fig. 4D, E,F). Additionally, the expression of Ki67 in tumor tissues decreased following STC2 knockdown (Fig. 4G).

**Fig. 5:**
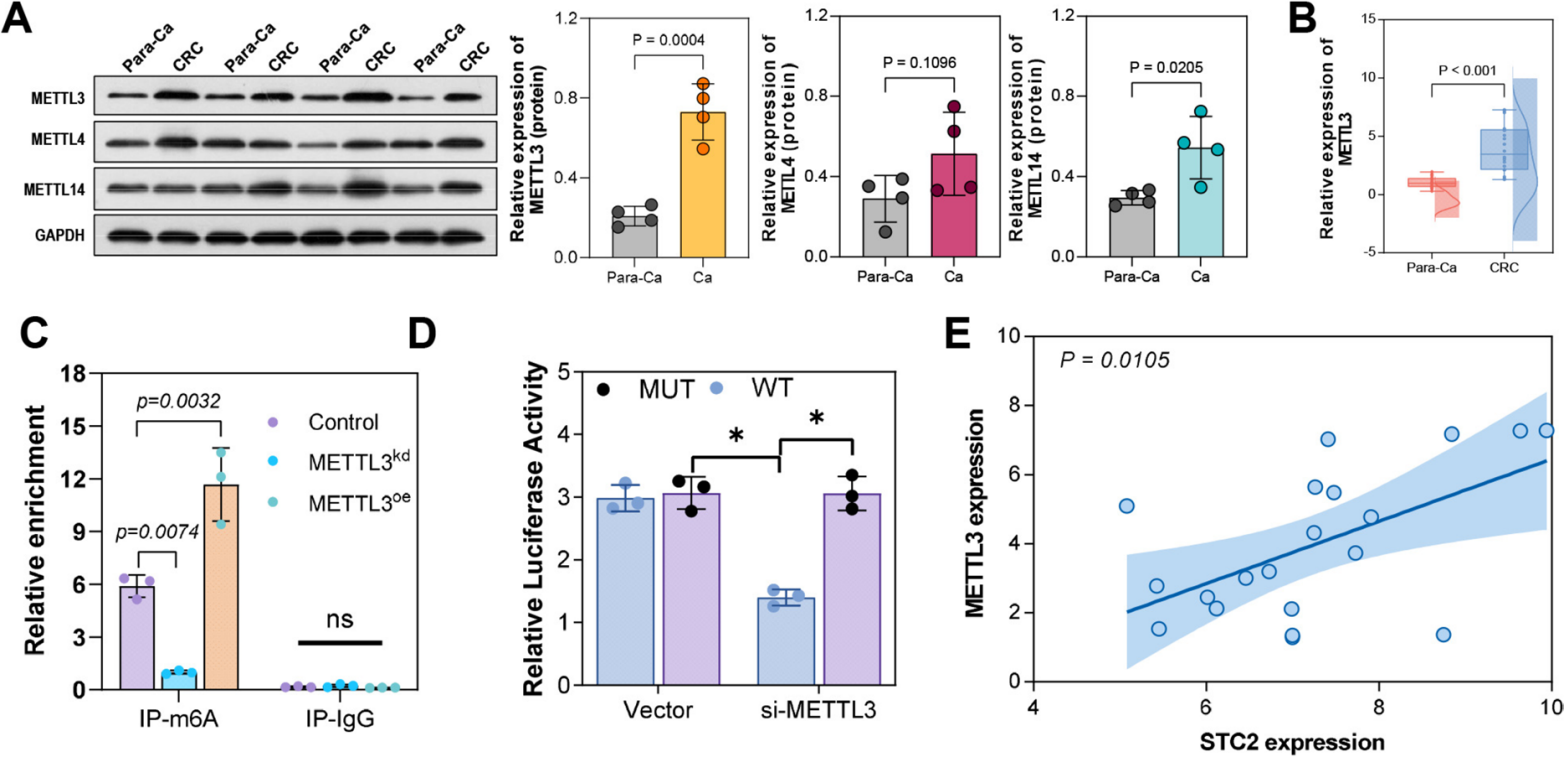
METTL3 positively regulates STC2 m6A and expression. (**A**, **B**) Expression of METTL3, METTL4 and METTL14 in tumor tissues by qPCR and Western blot. (**C**) Potential binding between METTL3 and STC2 m6A shown by RIP assay. (**D**) Decreased relative fluorescence intensity by METTL3 knockdown in dual-luciferase assay. (**E**) Positive correlation between STC2 and METTL3 expression levels by Pearson correlation analysis. *p < 0.05, ***p < 0.001

### METTL3 positively regulated STC2 m6 A and its expression

Previously, high methylation of STC2 (mRNA) was discovered in the experimental results. In this section, we mainly investigated the regulatory mechanisms of STC2 m6 A. Figure 5A showed that, in tumor tissues, methyltransferase METTL3 displayed a stable upregulation trend when compared to METTL4 and METTL14 (Fig. 5A, B). Moreover, the RIP assay indicated potential binding between METTL3 and STC2 m6 A (Fig. 5C). In the dual-luciferase assay, knockdown of METTL3 significantly decreased the relative fluorescence intensity (Fig. 5D). As shown in Fig. 5E, there was a significant positive correlation between the expression levels of STC2 and METTL3. Consequently, it can be concluded that STC2 m6 A is mainly regulated by METTL3.

### METTL3 silencing suppressed CRC cell proliferation and ERS by downregulating STC2

**Fig. 6:**
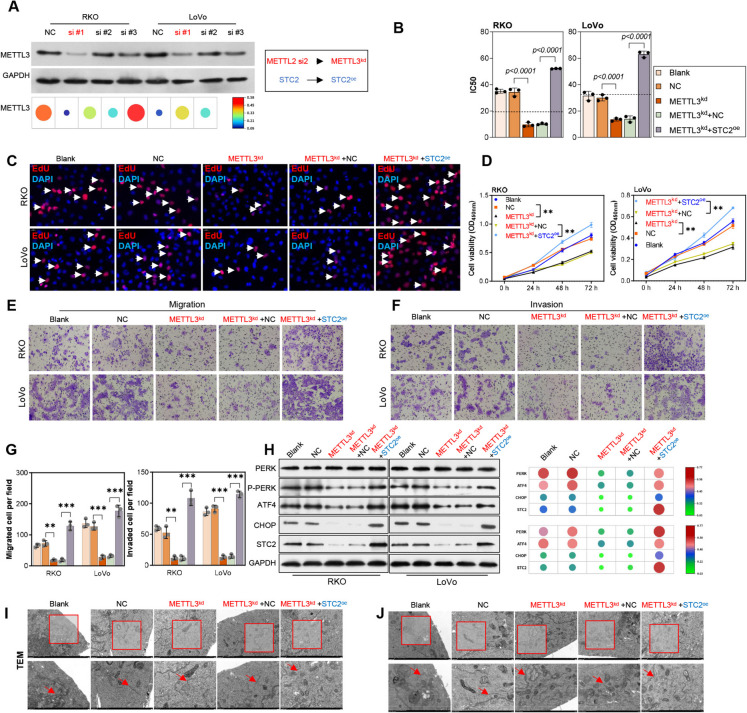
METTL3 silencing suppresses CRC cell proliferation and ERS by downregulating STC2. (**A**) siRNA #1 exhibited highest knockdown efficiency for METTL3 by Western blot. (**B**) METTL3 knockdown reduced IC50 of 5-FU in resistant cell lines, effect reversed by STC2 overexpression. (**C**-**D**) EdU staining and CCK-8 was used to detect cell proliferation. (**E**–**G**) Transwell assay showing cell migration and invasion. (**H**)The level of ERS related proteins were detected by Western blot. (**I**, **J**) Mitochondrial morphology was observed by transmission electron microscopy. **p < 0.01, ***p < 0.001

To further explore the biological role of METTL3-mediated regulation of STC2 in CRC drug resistance, this study employed siRNA targeting METTL3 in combination with STC2 overexpression. As shown in Fig. 6A, siRNA #1 exhibited the highest knockdown efficiency and was therefore selected for subsequent experiments **(**Fig. 6A**)**. According to Fig. 6B, METTL3 knockdown significantly reduced the IC50 of 5-FU in 5-FU resistant cell lines. However, this effect of METTL3 was reversed by STC2 overexpression (Fig. 6B). Afterwards, CCK-8 assay and EdU staining showed that cell proliferation was significantly suppressed by METTL3 knockdown. While, STC2 overexpression treatment significantly elevated the cell proliferation in 5-FU resistant cell lines (Fig. 6C, D). Transwell assays showed that METTL3 knockdown inhibited cell migration and invasion, whereas STC2 overexpression reversed this inhibition (Fig. 6E, F, G). To elucidate the regulatory role of METTL3/STC2 on ERS, we evaluated ERS in resistant cell lines after METTL3 knockdown using Western blot and transmission electron microscopy. The results showed that METTL3 knockdown significantly inhibited ERS in the cells, as indicated by the downregulation of p-PERK and ATF4 expression levels and the normalization of mitochondrial morphology. However, upon STC2 overexpression, the expression levels of p-PERK and ATF4 were increased, accompanied by swollen and fragmented mitochondria (Figs. 6H, I, J).

### METTL3 silencing promotes DNA damage in CRC cells and inhibits oxidative stress by down-regulating STC2

**Fig. 7:**
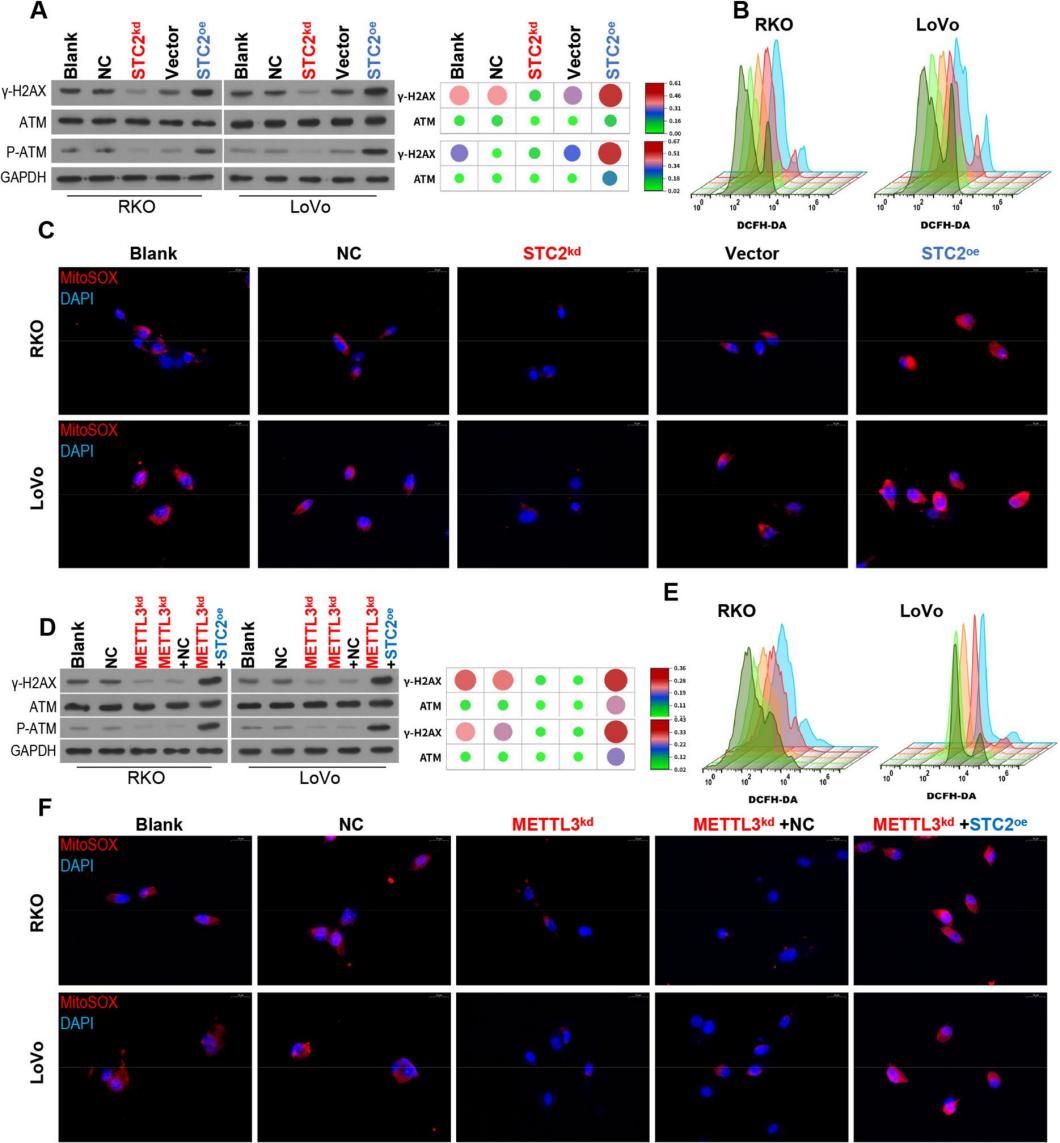
METTL3 silencing promotes DNA damage in CRC cells and inhibits oxidative stress by down-regulating STC2. (**A**) STC2 knockdown or STC2 overexpression plasmids were transfected into RKO and LoVo cells to detect the protein expression levels of γ-H2 AX, ATM and p-ATM by Western blot. (**B**) ROS levels were measured by flow cytometry. (**C**) Immunofluorescence assay to determine the fluorescence intensity of MitoSOX (**D**) RKO and LoVo cells were knocked down with METTL3 expression and transfected with STC2 overexpression plasmid. Western blot was used to detect the protein expression levels of γ-H2 AX, ATM and p-ATM. (**E**) ROS levels were measured by flow cytometry. (**F**) The fluorescence intensity of MitoSOX was detected by immunofluorescence assay, and the state of intracellular oxidative stress was evaluated

To further explore the role of STC2 and METTL3 in DNA damage resistance and oxidative stress in CRC cells. First, we transfected CRC cells with STC2 knockdown or STC2 overexpression plasmids. Western blot results showed that the expression levels of DNA damage response related proteins γ-H2 AX and p-ATM were significantly decreased after STC2 knockdown, which was opposite when STC2 was overexpressed (Fig. 7A). Meanwhile, results showed that ROS level and mitochondrial oxidative stress level were decreased in CRC cells after STC2 knockdown, while overexpression of STC2 had the opposite effect (Fig. 7B, C). To further explore the relationship between METTL3 and STC2 on cell damage, CRC cells were transfected with METTL3 knockdown plasmid and STC2 overexpression plasmid. Western blot showed that the expression of γ-H2 AX and p-ATM was decreased after METTL3 knockdown, while the expression of γ-H2 AX and p-ATM was significantly reversed after STC2 overexpression plasmid transfection (Fig. 7D). In addition, ROS level and mitochondrial oxidative stress were decreased in CRC cells after METTL3 knockdown. After co-transfection of STC2 overexpression plasmid, the decrease of ROS level and mitochondrial oxidative stress caused by METTL3 knockdown was significantly reversed (Fig. 7E, F). In summary, METTL3 silencing down-regulates STC2 expression, reduces the resistance of CRC cells to DNA damage, and reduces endoplasmic reticulum stress and oxidative stress.

### METTL3 leads to increased KRAS G12 and G13 mutations by promoting STC2 expression

**Fig. 8:**
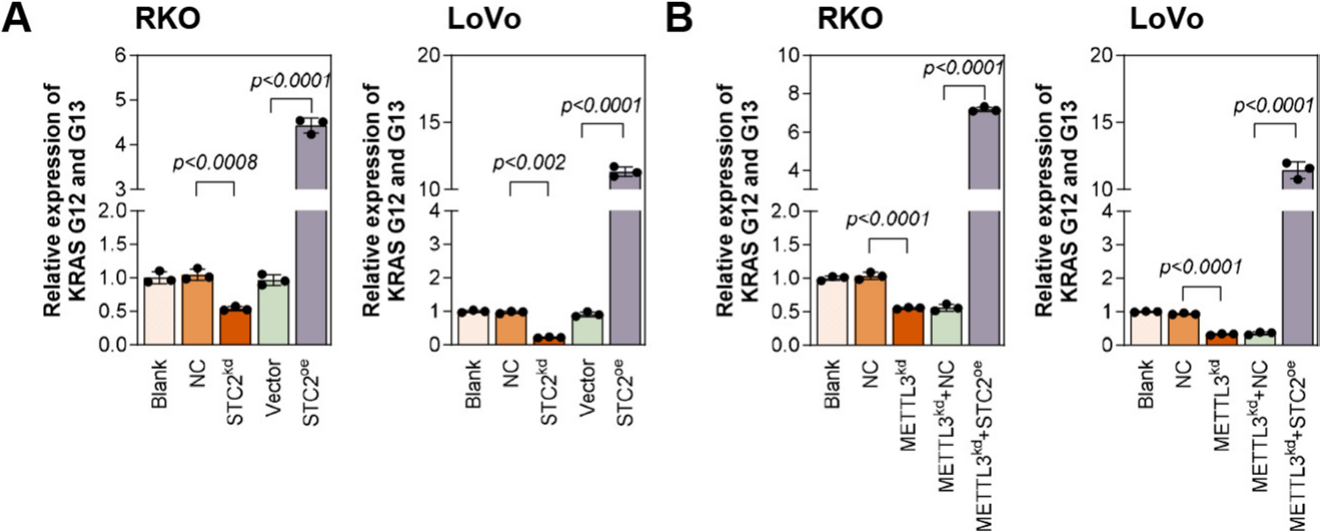
METTL3 increases KRAS G12 and G13 mutations by promoting STC2 expression (**A**) Cultured CRC cells transfected with STC2 knockdown and overexpression plasmids. qPCR was used to detect the expression levels of KRAS G12 and G13 mutations in the cells. (**B**) RKO and LoVo cells were knocked down for METTL3 expression and transfected with STC2 overexpression plasmid. qPCR was used to detect the expression levels of KRAS G12 and G13 mutations in the cells. **p < 0.01, ***p < 0.001

It has been found that KRAS gene mutation is a common genetic change in many cancers. G12 and G13 are two common mutation sites in the KRAS gene, and these mutations lead to persistent activation of the KRAS protein, thereby promoting cell proliferation and tumor formation. In our study, we found that the level of KRAS G12 and G13 mutation was significantly reduced in cells transfected with STC2 knockdown plasmid, while the opposite result was observed when transfected with STC2 overexpression plasmid (Fig. 8A). In addition, knockdown of METTL3 expression reduced the level of KRAS G12 and G13 mutations, while co-transfection of STC2 overexpression plasmid significantly increased the expression of KRAS G12 and G13 mutations (Fig. 8B). In summary, METTL3 promotes KRAS G12 and G13 mutations by promoting STC2 expression.

### METTL3/STC2 axis promoted sensitivity to 5-FU and tumor metastasis in vivo

**Fig. 9:**
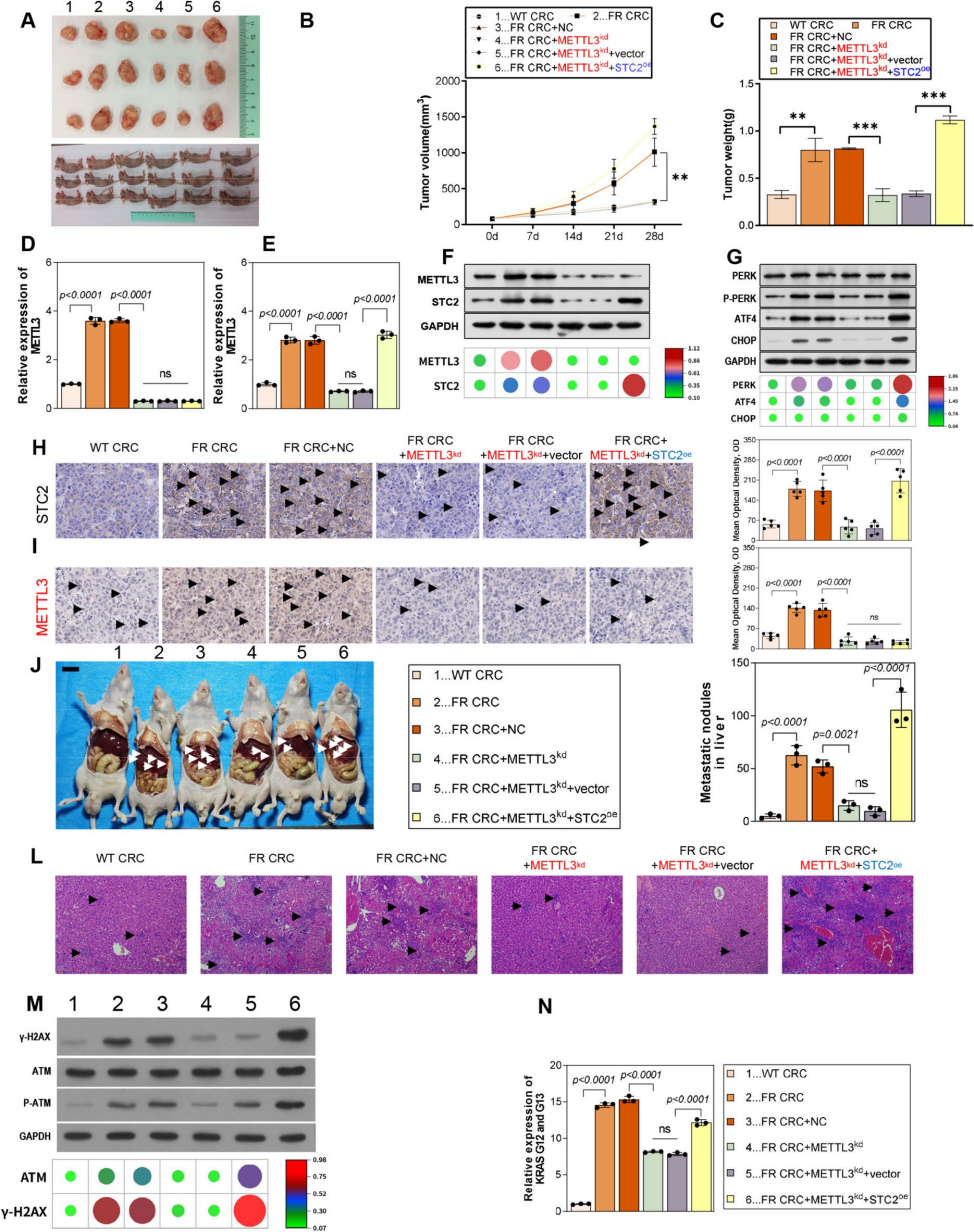
METTL3/STC2 axis influences sensitivity to 5-FU and tumor metastasis in vivo. (**A**) Representative images of 5-FU-resistant cells transfected with METTL3 knockdown plasmid and STC2 overexpressing plasmid in nude mice. (**B**) Tumor growth curve shows METTL3 knockdown enhanced 5-FU inhibition of tumor growth, reversed by STC2 overexpression. (**C**) In contrast, METTL3 knockdown and STC2 overexpression of xenografts resulted in tumor weight. (**D**-**F**) qPCR and Western blot confirming METTL3 and STC2 expression levels in resistant strains. (**G**) Western blot showing changes in PERK and ATF4 expression with METTL3 inhibition and STC2 overexpression. (**H**, **I**) The levels of STC2 and Ki67 were determined by Immunohistochemistry. (**J**, **K**) Representative diagram of liver metastasis model and quantitative analysis diagram of metastatic nodules in nude mice. (**L**) H&E staining image of tumor tissue. (**M**) The protein expression levels of γ-H2 AX, ATM and p-ATM were detected by Western blot. (**N**) The mutant expression levels of KRAS G12 and G13 were detected by qPCR. *p < 0.05, **p < 0.01, ***p < 0.001

To examine the response of METTL3/STC2 to 5-FU in vivo tumor models, this section manipulated the expression of METTL3 and STC2 in 5-FU-resistant LoVo cells. As illustrated in Fig. 9A, wild-type LoVo cells displayed higher sensitivity compared to the resistant strain. Knockdown of METTL3 greatly enhanced the inhibitory effect of 5-FU on tumor growth, which was notably hindered upon STC2 overexpression (Fig. 9A-C). qPCR, Western blot and Immunohistochemistry results identified high expression of METTL3 and STC2 in the resistant strain, with METTL3 knockdown leading to downregulation of STC2 expression (Fig. 9D-F, H). Western blot analysis indicated that inhibition of METTL3 expression resulted in downregulation of PERK and ATF4 expressions, which were restored upon STC2 overexpression (Fig. 9F, I).

In a subcutaneous tumor-bearing mouse model, experimental results showed that METTL3 knockdown significantly inhibited tumor metastasis to the liver. However, this inhibitory effect was significantly reversed by STC2 overexpression (Figs. 9J-L). In addition, overexpression of STC2 reversed the downregulation of γ-H2 AX and p-ATM expression and the reduction of KRAS G12 and G13 mutations caused by METTL3 knockdown (Fig. 9M, N). In conclusion, in vivo experiments further verified that METTL3 promoted DNA damage by up-regulating STC2 expression, leading to mutations at G12 and G13 sites of KRAS, and promoting the progression of CRC cells.

## Discussion

**Fig. 10:**
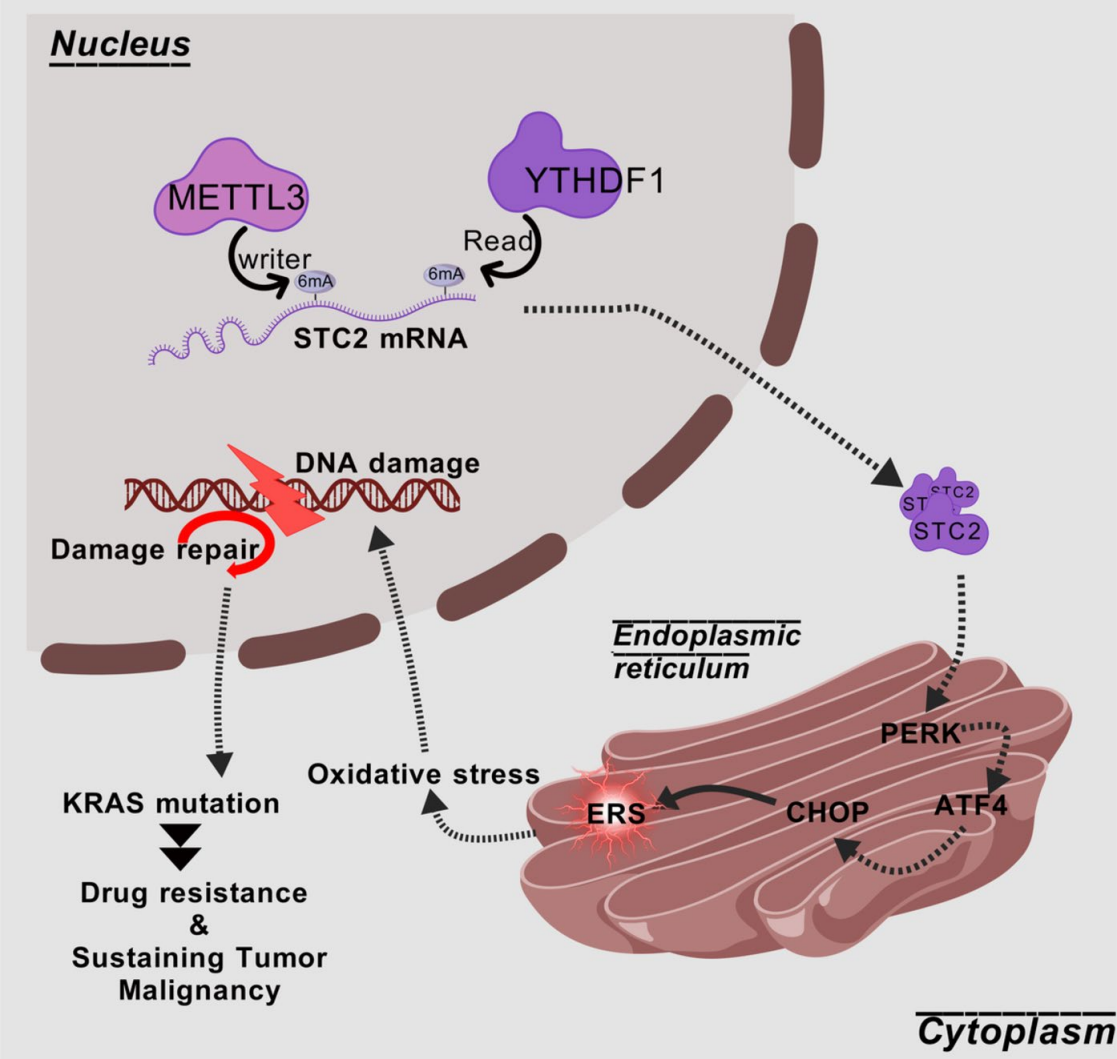
The graphic abstract of this study

Although surgery, chemotherapy, radiation therapy and molecular therapy are available for CRC therapy, the survival rate of CRC patients remains low (Biller and Schrag 2021). Drug resistance significantly limits the therapeutic efficacy of key CRC chemotherapies like 5-FU. This study found METTL3 positively regulates STC2 via m6 A modification in CRC. The METTL3/STC2 axis promotes 5-FU resistance, proliferation, ERS, oxidative stress, and KRAS G12/G13 mutations. This study aimed to investigate the METTL3/STC2 axis's role in CRC 5-FU resistance and progression via m6 A modification Fig. 10.

N6-methyladenosine (m6 A) modification is a critical epigenetic regulator, exhibiting heterogeneous and context-dependent roles across various cancers by influencing diverse cellular pathways (Lin et al. 2024). In digestive system malignancies specifically, dysregulation of m6 A homeostasis frequently contributes to tumor progression. For instance, elevated expression of the m6 A'writer'METTL3 in CRC is linked to poor prognosis and promotes malignancy through targets such as CRB3 and the Hippo pathway (Pan et al. 2022). Conversely, the downregulation of the m6 A'eraser'ALKBH5 in gastric cancer also enhances malignant phenotypes; the resulting hypermethylation and subsequent dysregulation of target transcripts like WRAP53 are associated with increased GC cell proliferation and invasion, potentially involving the PI3 K/Akt/mTOR pathway (Zheng et al. 2025). m6 A modifications modulated tumor resistance by affecting drug metabolizing enzymes, multidrug effector transporters, and anti-cancer drug targets (Zhuang et al. 2023). Studies also testified that m6 A modification is essential for cancer development, progression, metastasis, drug resistance and recurrence (Xue et al. 2024; Dai et al. 2025). In our study, we also analyzed the characteristics of m6 A modification in 5-FU-sensitive and resistant CRC tissues through MeRIP-seq analysis. Results displayed that m6 A peak was mainly enriched in CDS in exon region, which is consistent with previous findings on m6 A in eukaryotic cells (Zou, Shen et al. 2023). More importantly, we screened for numerous hypermethylated and highly expressed genes, mainly STC2, KLHL13, CACNG8, and KCNMB3. Through validation, we proved that STC2 was upregulated in CRC patients and 5-FU resistant CRC cells.

Stanniocalcin (STC) is a specific protein transcribed and translated from the STC gene, which is a glycoprotein hormone involved in the regulation of calcium and phosphorus homeostasis. STC2 is a member of the STC family and is widely distributed in various parts of the body, such as bone, pancreas and colon (Qie and Sang 2022). In addition to the regulation of calcium and phosphorus metabolism, STC2 also regulates skeletal development, oxidative stress, angiogenesis and other physiological and pathological processes (Joshi 2020). Besides, several studies indicated abnormal expression of STC2 gene in tumor tissues, such as rectal cancer (Huang et al. 2021), gastric cancer (Ke et al. 2019), pancreatic cancer (Lin et al. 2019), etc. Furthermore, recent evidence links high STC2 expression in CRC tissues to metastasis and poor survival outcomes (Ke et al. 2019). It has also been demonstrated that STC2 could accelerate CRC cell proliferation, epithelial mesenchymal transition and migration, and activates the Wnt/β-catenin pathway (Chen et al. 2016; Ke et al. 2019). However, it is not clear whether STC2 can affect 5-FU-resistant CRC cells-related functions. In our study, we revealed for the first time that STC2 could enhance the proliferation and ERS of 5-FU-resistant CRC cells. Meanwhile, our data denoted that STC2 silencing also could reduce the tumor growth of 5-FU -resistant CRC mice. Thus, we verified that STC2 silencing has obvious inhibitory effect on the process of 5-FU resistant CRC.

Abnormal expression of METTL3 is closely related to the occurrence and development of various cancers. By mediating the m6 A modification of mRNA, METTL3 promotes tumor drug resistance and metastasis (Shao, Han et al. 2024). As demonstrated in rectal adenocarcinoma, silencing METTL3 expression markedly reversed 5-FU resistance in HCT-8 cells (Li et al. 2022). METTL3 promotes 5-FU chemoresistance in colorectal cancer by increasing TRAP1 expression through N6-methyladenosine (m6 A)-dependent stabilization of its mRNA, thereby identifying the METTL3/TRAP1 axis as a key regulator of drug sensitivity (Kang et al. 2025). Besides, METTL3 promotes colorectal cancer metastasis by leveraging its N6-methyladenosine (m6 A) modification function to stabilize and upregulate REG1α, consequently activating the β-catenin/MYC/LDHA axis to enhance glycolysis (Zhou et al. 2023). Therefore, knockdown of METTL3 potentially attenuated cancer metastasis and drug resistance. For instance, METTL3 knockdown promotes sensitivity of glioma stem cells via DNA repair (Shi et al. 2023). This study indicated that METTL3 can positively regulate STC2 and mediate ERS. METTL3 silencing reduces tumor growth in 5-FU-resistant CRC xenografts by reducing KRAS G12 and G13 mutations through STC2.

Conventional cancer therapies, such as radiotherapy and chemotherapy (e.g., 5-FU), frequently impose substantial stress on tumor cells, inducing pronounced endoplasmic reticulum stress (ERS) (Chen and Cubillos-Ruiz 2021). This treatment-induced ERS disrupts cellular homeostasis, triggers the unfolded protein response (UPR), and significantly elevates the production of reactive oxygen species (ROS), culminating in severe oxidative stress (Chen and Cubillos-Ruiz 2021; Yuan et al. 2025a, b). Persistent oxidative stress can directly attack DNA, inflicting damage. Concurrently, ERS itself can activate key proteins within the DNA damage response (DDR) pathway, such as modulating the phosphorylation of ATM (Murray et al. 2024). Consequently, the accumulation of treatment-induced ERS and ROS collectively promotes the onset of DNA damage. This is typically manifested by the upregulation of DDR markers, such as γ-H2 AX and phosphorylated ATM (p-ATM), which normally serve to direct cells towards repair or apoptosis (Yuan et al. 2024). Indeed, studies (e.g., in testicular cancer) have demonstrated that radiotherapy and chemotherapy can upregulate the expression of γ-H2 AX and ATM through the accumulation of ERS and ROS (Lee et al. 2023). However, in drug-resistant tumor cells, the activation of this DDR signaling does not invariably lead to effective cellular clearance. Instead, cells may exploit enhanced or dysregulated DDR mechanisms to tolerate the damage and ensure survival (Murray et al. 2024). Critically, this persistent and potentially imprecise DNA damage repair process enhances genomic instability, consequently increasing the risk of acquiring crucial gene mutations, such as those in KRAS G12 and G13 (Li, Wang et al. 2022a, b). Once generated and selected, these mutations can bestow significant survival and proliferative advantages upon the cells (Gu et al. 2023), driving tumor progression. Our present study elucidates that in colorectal cancer, METTL3-mediated m6 A modification upregulates STC2 expression. This, in turn, drives a cascade involving ERS, oxidative stress, alterations in the DNA damage response (reflected by changes in p-ATM/γ-H2 AX levels), and increased KRAS mutations, ultimately promoting cellular resistance to 5-FU and enhancing metastatic potential.

Notably, KRAS mutations are key drivers in multiple solid tumors (McDaid et al. 2024; Varghese et al. 2025). Emerging evidence indicates that KRAS-mutant tumors resist chemotherapy by activating the unfolded protein response (UPR) and oxidative stress (Yang et al. 2019), a pathway strikingly aligned with the STC2-induced ERS-ROS axis identified here. Interesting, a newly report have found that KRAS mutations drive platinum resistance in non-small cell lung cancer by dysregulating m6 A modification; specifically, KRAS signaling inhibits the demethylase ALKBH5, leading to increased m6 A-mediated stabilization of DNA damage repair gene mRNAs (DDB2/XPC), which enhances repair capacity to overcome drug-induced DNA damage and confers resistance (Yu et al. 2025). Furthermore, the pan-cancer upregulation of METTL3 and STC2 has been well-documented (Qie and Sang 2022; Qi et al. 2023). Thus, the METTL3/STC2 axis may exert pro-resistance effects across cancers via the ERS-KRAS mutation pathway. Future studies should validate the universality of this mechanism in pan-cancer contexts and explore combination therapies targeting METTL3 or STC2 alongside KRAS inhibitors (e.g., Sotorasib) to address cross-cancer chemotherapy resistance. However, therapeutic strategies targeting METTLs in oncology remain in nascent stages. For instance, STC-15, a METTL3 inhibitor currently under clinical investigation, has demonstrated preliminary disease control in early trials (Moser et al. 2024). Additionally, combining METTL3 inhibition with miRNA-based therapies may provide a multi-layered epigenetic intervention, as miRNA-m6 A crosstalk is emerging as a key regulator of oncogenic pathways (Panagal et al. 2018; Qian et al. 2023). In contrast, STC2-targeted approaches are still confined to preclinical exploration. Future development of synergistic therapies will necessitate overcoming critical challenges such as tumor-specific drug delivery and biomarker-driven patient stratification.

## Limitations

This study has elucidated crucial aspects of the METTL3/STC2 axis in colorectal cancer progression and drug resistance, utilizing established in vitro cell lines and xenograft models. While these systems are invaluable for dissecting molecular mechanisms, it is recognized that they provide a simplified view compared to the complex tumor ecosystem in vivo. Future investigations employing more sophisticated models, such as patient-derived organoids or syngeneic models that incorporate the interplay with the host immune system, could offer a more complete picture of how this axis functions within the physiological tumor microenvironment. Furthermore, while we have demonstrated strong associations between the METTL3/STC2 axis, endoplasmic reticulum stress, oxidative stress, DNA damage, and KRAS mutations, the intricate molecular cascade precisely linking STC2's role in stress responses to the emergence or selection of specific KRAS mutational variants presents a compelling area for further detailed mechanistic dissection. Translational impact is underscored by our initial clinical observations, and validating these preclinical findings in larger, diverse patient cohorts, correlating METTL3/STC2 expression with KRAS mutation status and treatment response outcomes, would be a pivotal next step towards clinical application.

## Conclusion

In summary, the results suggest that STC2, a gene that is upregulated and hypermethylated in CRC tissues, promotes ERS and oxidative stress through the m6 A modification mechanism regulated by METTL3, and increases KRAS G12 and G13 mutations. Further promotes cell proliferation and resistance to 5-FU. Importantly, the knockdown of STC2 not only increased the sensitivity of CRC cells to 5-FU but also significantly inhibited tumor metastasis to the liver. These findings suggest STC2 as a potential therapeutic target for overcoming drug resistance and preventing metastasis and reduced KRAS G12 and G13 mutations in CRC treatment. This underscores the importance of understanding the molecular mechanisms underlying drug resistance and metastasis and offers a promising direction for enhancing the efficacy of CRC therapies.

## Supplementary Information

Below is the link to the electronic supplementary material.

Supplementary file1 (DOCX 24 KB)

ESM 2 (PNG 562 KB)

High Resolution Image (TIF 2.21 MB)

## Data Availability

Data are available from the corresponding author on reasonable request.

## Abbreviations

*ANOVA*: Analysis of variance
*ATF4/6*: Activating transcription factor 4/6
*ATM*: Ataxia telangiectasia mutated
*BSA*: Bovine serum albumin
*CCK-8*: Cell counting Kit-8
*CDS*: Coding sequence
*COAD*: Colorectal adenocarcinoma
*CRC*: Colorectal cancer
*DAPI*: 4′,6-Diamidino-2-phenylindole
*DCFH-DA*: 2’,7’-Dichlorofluorescein diacetate
*EdU*: 5-Ethynyl-2’-deoxyuridine
*ERS*: Endoplasmic Reticulum Stress
*5-FU*: 5-Fluorouracil
*FBS*: Fetal bovine serum
*FC*: Fold change
*GEPIA*: Gene expression profiling interactive analysis
*GO*: Gene ontology
*H&E*: Hematoxylin and eosin
*IC50*: Half maximal inhibitory concentration
*IGV*: Integrative genomics viewer
*IHC*: Immunohistochemistry
*m6 A*: N6-methyladenosine
*MeRIP-seq*: Methylated RNA immunoprecipitation sequencing
*NC*: Negative control
*PDAC*: Pancreatic ductal adenocarcinoma
*READ*: Rectal adenocarcinoma
*ROS*: Reactive oxygen species
*siRNA*: Small interfering RNA
*SPF*: Specific pathogen-free
*SRAMP*: Sequence-based RNA adenosine methylation site predictor
*TEM*: Transmission electron microscope
*UPR*: Unfolded protein response
*UTR*: Untranslated region
