# Supplementary material for: Dissecting the METTL3/STC2 axis in colorectal cancer: implications for drug resistance and metastasis
Source: Cell Biol Toxicol. 2025 Jun 10;41(1):100. doi: 10.1007/s10565-025-10043-5 (PMC12152045; doi:10.1007/s10565-025-10043-5)
Supplement: Supplementary file 1 — Supplementary file1 (DOCX 24 KB) [file 10565_2025_10043_MOESM1_ESM.docx]

**Methods**

*Cell culture*

RKO and SW620 cell lines were purchased from National Institute of Cell Resources (Beijing, China). RKO cells were grown in MEM medium (Gibco; cat. no. 41500034); Lovo cells were incubated in Dulbecco’s Modified Eagle Medium (Gibco, cat. no. 12800017). Both types of mediums included 10% fetal bovine serum (FBS, Sigma). The cells were cultured in a cell incubator containing saturated humidity, 5% CO_2_ and 37°C. In this study, STC2 expressions in LoVo and RKO were higher and were chosen for further experiments.

*Cell transfection*

STC2 siRNAs (siRNA#1, #2, and #3) and negative control (NC) were acquired from GenePharma (Suzhou, China). STC2 overexpression plasmid and vector were acquired from Thermo Fisher Scientific. RKO and LOVO cells or 5-FU resistant cells (1×10^5^cells/well) were evenly placed into a 6-well plate and cultured to 80% fusion. The cells were transfected with siRNAs or STC2 overexpression plasmid using Lipofectamine^TM^ 3000 (Invitrogen) on account of the specification.

*EdU (5-Ethynyl-2’-deoxyuridine) staining*

EdU staining was used to measure cell proliferation of CRC. Cells were gently inoculated in 24-well plates (8×10^3^ cells/well) and processed. Based on the instructions of EdU kit, cells in each well were dealt with 100 μL of medium containing EdU probe (50 pmol/L) for 2 h at 37℃. After washing, the cells were fixed by 4% paraformaldehyde. Cells labeled with EdU probe (red fluorescence) and Hoechst33342 (blue fluorescence) were counted under the microscope.

*CCK-8 (cell counting kit 8)*

CCK-8 assay was used to assess cell viability and proliferation. Briefly, each group of cells was collected, counted, and prepared in cell suspension (5×10^4^ cells/mL). Then 100 μL of cell suspension was equably inoculated into 96-well plates. After overnight incubation, cells in each well were processed for experimental purposes. After 24, 48, and 72 h, cells were incubated with CCK-8 (final concentration 10%) for 2 h. The absorbance values at 450 nm were tested using a microplate reader.

*Immunohistochemistry*

Paraffin-embedded sections were dewaxed, hydrated, washed twice with PBS, and closed with 3% H_2_O_2_ for 10 min. After washing with distilled water, antigen repair was conducted. After washing, sections were closed with BSA for 20 min, primary antibodies (STC2, Ki-67 or METTL3) overnight at 4°C, and biotinylated secondary antibody at 37°C for 20 min. After washing, sections were drop-wrapped with SABC at 37°C for 20 min. The sections were colored with DAB, redyed with hematoxylin for 2 min, differentiated by hydrochloric alcohol, dehydrated, transparent and sealed. Finally, the results were observed under the microscope.

*M6A-qPCR*

After transfection for 48h, total RNA (300μg) of cells was extracted for MeRIP-qPCR experiments. The MagnaMeRIP™ m6A kit (Millipore, USA) was selected and m6A RNA immunoprecipitation were performed by referring to the instructions. The final enriched m6A-modified RNA was verified and then subjected to RT-qPCR analysis.

*mRNA-sequencing and meRIP-sequencing*

Total RNA from the samples (N=3 per group), meeting the quality requirements, was used for sequencing library construction. Specifically, RNA-seq libraries (representing the input control for meRIP-seq and used for gene expression profiling) were prepared. The sequencing was performed on an Illumina Hiseq X Ten platform generating 150 bp paired-end reads. Raw data quality control was performed using Fastp (v0.20.0) to obtain clean reads. Clean reads were subsequently aligned to the human reference genome (GRCh38) using Hisat2 (v2.1.0). Uniquely mapped reads were retained. Gene expression levels were quantified using featureCounts based on read counts mapped to gene features. Differential gene expression analysis between comparison groups was then performed using the DESeq2 package in R. Genes were considered significantly differentially expressed if they met the criteria of an adjusted P-value (FDR) < 0.05 and an absolute log2(Fold Change) > 1.

Corresponding RNA-seq libraries served as the input control. Both IP and Input libraries were sequenced on an Illumina Hiseq X Ten platform (150 bp paired-end reads). Raw reads underwent quality control with Fastp (v0.20.0). Clean reads from both IP and Input samples were aligned to the human reference genome (GRCh38) using Hisat2 (v2.1.0), retaining uniquely mapped reads. Detection of m6A peaks was performed using MeTDiff software by comparing IP versus Input signals. Differential m6A peaks/modification levels between comparison groups were subsequently identified using MeTDiff, based on screening criteria of P-value < 0.05 and |log2(Fold Change) | > 1. Enriched motif discovery within peaks was performed using MEME-ChIP (v5.0.5). The distribution of m6A peaks across transcript regions was analyzed using the R package Guitar (v1.1.1.18). Association analysis comparing differential m6A modification (from meRIP-seq) and differential gene expression (from mRNA-seq) was visualized using ggplot software.

*RT-qPCR (Reverse Transcription Quantitative Polymerase Chain Reaction)*

TRIzol (Invitrogen, MA, USA) was applied to extract total RNA from the tissues and transfected cells. And total RNA was reversely transcribed into cDNA using reverse transcription kit (Takara, Tokyo, Japan). RT-qPCR was performed using cDNA template using SYBR Green qPCR Master Mix (DBI Bioscience) on ABI 7500 real-time PCR machine (ABI, ViiA7). The qPCR thermal cycling protocol involved an initial denaturation at 95 ^o^C for 1 min, followed by 40 cycles of 95 ^o^C for 5 sec and 60 ^o^C for 30 sec, concluding with a melt curve analysis from 60 ^o^C to 95^o^C. Gene expression was calculated by 2^-ΔΔCt^ with GAPDH as the internal parameter. The primers used in this experiment were shown as Table 1.

*Transmission electron microscope (TEM)*

After treatment, the cells were collected and fixed with 2.5% glutaraldehyde at 4°C for 2 h. After washing, cells were fixed with 2% osmium tetroxide at 4°C for 3 h. After washing, cells were dehydrated in 30%-50%-70%-90%-95%-100% ethanol. Then the cells were treated with acetone and permeabilized with a mixture of endone and epoxy resin. The cells were embedded successively by 35°C for 12 h, 45°C for 12 h, and finally 60°C for 24h. Ultra-thin sections and conventional electron microscopic staining were performed. Then Ultrathin sections and routine staining were performed, and the ultrastructure was observed under TEM.

*Western blot*

The groups of cells and tissues were collected, and the tissue homogenates and cells were lysed with RIPA (Beyotime, China) on ice for 30 min. After centrifugation, the supernatant was collected. Then the protein concentration was examined by BCA kit. 20 μg of proteins were subjected to 10% SDS-PAGE electrophoresis. After electrophoretic separation, the proteins were transferred to PVDF membrane (Millipore) and then closed with 5% skim milk powder for 1 h. After washing, the PVDF membrane was incubated with diluted primary antibody (Abcam, 1:1000) at 4°C overnight, and then secondary antibody (Abcam, 1:2000) for 1.5 h. ECL reagent (Millipore)was added to observe the protein bands.

*Reactive oxygen species (ROS) production*

The 2’,7’-dichlorofluorescein diacetate (DCFH-DA) assay kit (Enzo Life Sciences, Farmingdale, NY, USA) to measure the intracellular ROS (Reactive Oxygen Species) levels following the instructions provided by the manufacturer. In summary, the cells were detached and converted into a single-cell suspension through trypsin treatment. After being rinsed three times with phosphate-buffered saline (PBS), the cells were exposed to 10 μM DCFH-DA and incubated at 37°C for a duration of 20 minutes. Following this incubation, the cells were re-suspended in PBS, and the ROS content was then evaluated using a BD Biosciences FACSCalibur flow cytometer.

*MitoSOX Immunofluorescence assay*

RKO and LoVo cells were inoculated separately in a cell imaging dish with a 35 mm glass base. After 15 minutes of culture, the cells were immobilized with 4% paraformaldehyde and permeated in a phosphate buffer containing 0.3% Triton X-100 for 15 minutes. The cells were then enclosed on PBS with 5% cornerbacks for 1 hour and subsequently incubated with MitoSOX red dye overnight at 4 °C. The next day, the cells were incubated with Alexa Fluor® 647 labeled goat anti-rabbit immunoglobulin secondary antibody for 2 hours. Eventually, the cells were properly loaded and DAPI fluorescence images were obtained using a light microscope (Leica DM 2500, Germany) at 200x magnification.
