# Supplementary figures and images for: Dissecting the METTL3/STC2 axis in colorectal cancer: implications for drug resistance and metastasis
Source: Cell Biol Toxicol. 2025 Jun 10;41(1):100. doi: 10.1007/s10565-025-10043-5 (PMC12152045; doi:10.1007/s10565-025-10043-5)

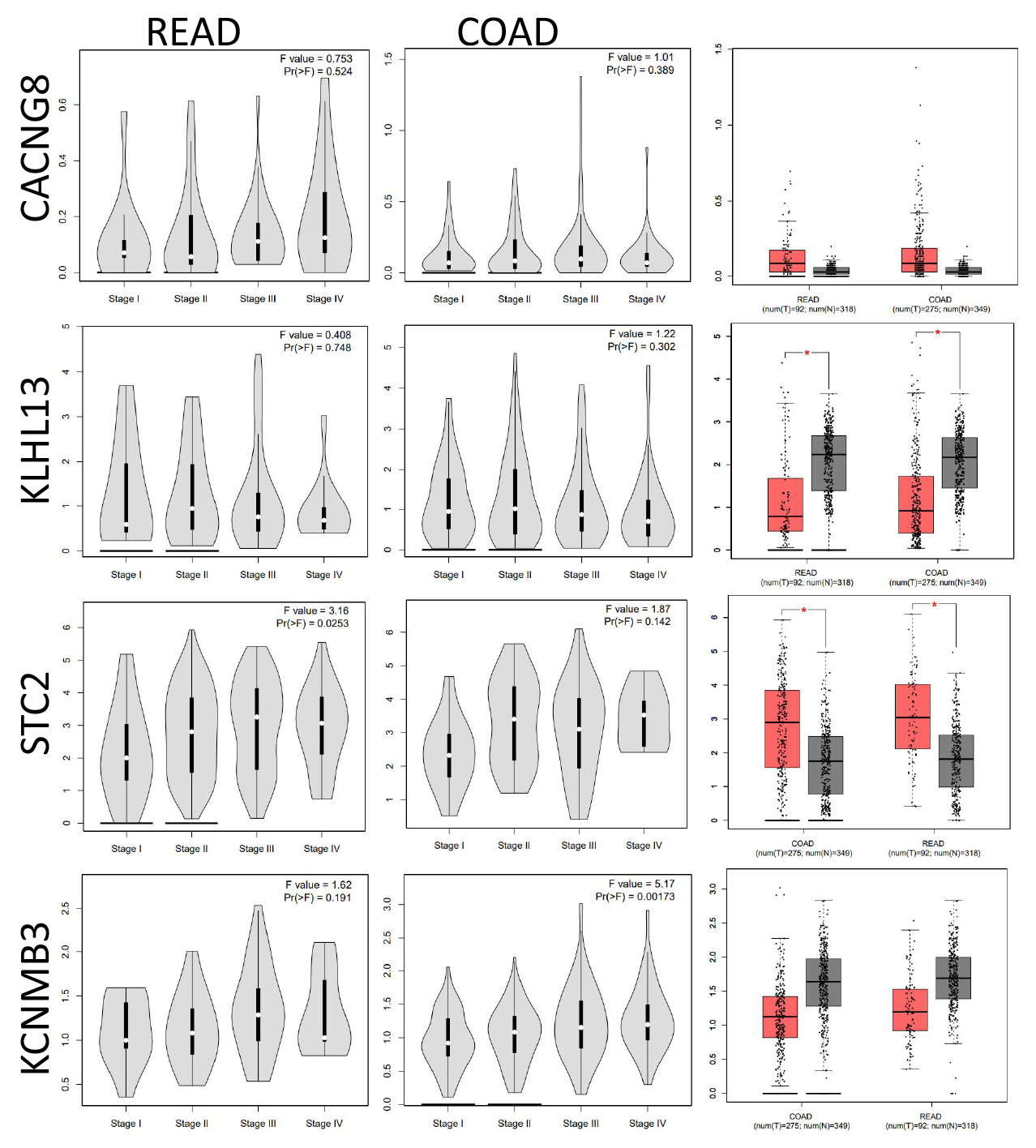

Supplement: Supplementary file 2 — (PNG 562 KB) [file 10565_2025_10043_Fig11_ESM.png]

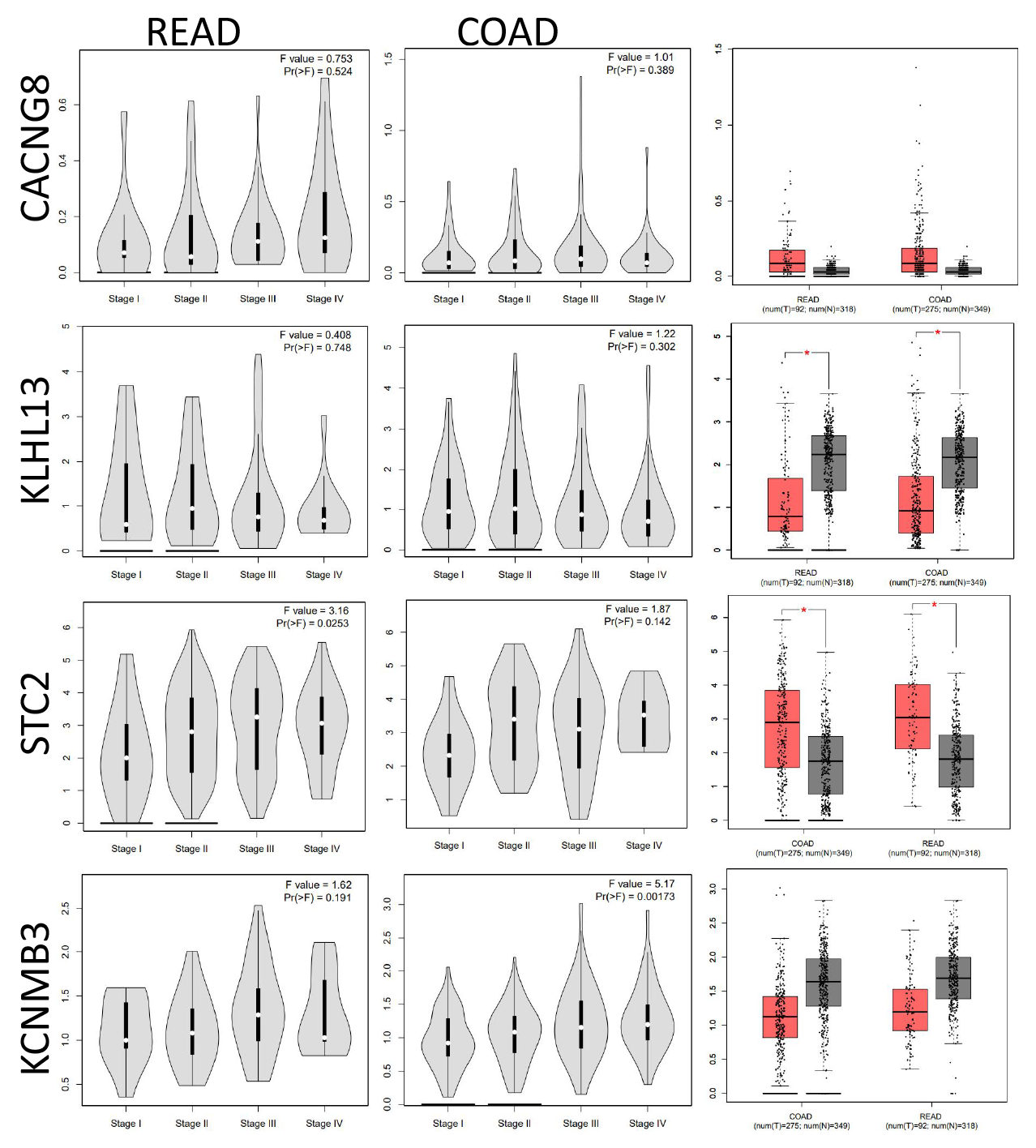

Supplement: Supplementary file 3 — High Resolution Image (TIF 2.21 MB) [file 10565_2025_10043_MOESM2_ESM.tif]
